# iGRLDTI: an improved graph representation learning method for predicting drug–target interactions over heterogeneous biological information network

**DOI:** 10.1093/bioinformatics/btad451

**Published:** 2023-07-28

**Authors:** Bo-Wei Zhao, Xiao-Rui Su, Peng-Wei Hu, Yu-An Huang, Zhu-Hong You, Lun Hu

**Affiliations:** The Xinjiang Technical Institute of Physics and Chemistry, Chinese Academy of Sciences, Urumqi 830011, China; University of Chinese Academy of Sciences, Beijing 100049, China; Xinjiang Laboratory of Minority Speech and Language Information Processing, Urumqi 830011, China; The Xinjiang Technical Institute of Physics and Chemistry, Chinese Academy of Sciences, Urumqi 830011, China; University of Chinese Academy of Sciences, Beijing 100049, China; Xinjiang Laboratory of Minority Speech and Language Information Processing, Urumqi 830011, China; The Xinjiang Technical Institute of Physics and Chemistry, Chinese Academy of Sciences, Urumqi 830011, China; University of Chinese Academy of Sciences, Beijing 100049, China; Xinjiang Laboratory of Minority Speech and Language Information Processing, Urumqi 830011, China; School of Computer Science, Northwestern Polytechnical University, Xi’an 710129, China; School of Computer Science, Northwestern Polytechnical University, Xi’an 710129, China; The Xinjiang Technical Institute of Physics and Chemistry, Chinese Academy of Sciences, Urumqi 830011, China; University of Chinese Academy of Sciences, Beijing 100049, China; Xinjiang Laboratory of Minority Speech and Language Information Processing, Urumqi 830011, China

## Abstract

**Motivation:**

The task of predicting drug–target interactions (DTIs) plays a significant role in facilitating the development of novel drug discovery. Compared with laboratory-based approaches, computational methods proposed for DTI prediction are preferred due to their high-efficiency and low-cost advantages. Recently, much attention has been attracted to apply different graph neural network (GNN) models to discover underlying DTIs from heterogeneous biological information network (HBIN). Although GNN-based prediction methods achieve better performance, they are prone to encounter the over-smoothing simulation when learning the latent representations of drugs and targets with their rich neighborhood information in HBIN, and thereby reduce the discriminative ability in DTI prediction.

**Results:**

In this work, an improved graph representation learning method, namely iGRLDTI, is proposed to address the above issue by better capturing more discriminative representations of drugs and targets in a latent feature space. Specifically, iGRLDTI first constructs an HBIN by integrating the biological knowledge of drugs and targets with their interactions. After that, it adopts a node-dependent local smoothing strategy to adaptively decide the propagation depth of each biomolecule in HBIN, thus significantly alleviating over-smoothing by enhancing the discriminative ability of feature representations of drugs and targets. Finally, a Gradient Boosting Decision Tree classifier is used by iGRLDTI to predict novel DTIs. Experimental results demonstrate that iGRLDTI yields better performance that several state-of-the-art computational methods on the benchmark dataset. Besides, our case study indicates that iGRLDTI can successfully identify novel DTIs with more distinguishable features of drugs and targets.

**Availability and implementation:**

Python codes and dataset are available at https://github.com/stevejobws/iGRLDTI/.

## 1 Introduction

A critical step in drug development is to validate the safety and efficacy of new drugs by discovering the active compound molecule interacting with target proteins before marketing (D’Souza *et al.* 2020). Hence, the identification of unknown drug–target interactions (DTIs) is of great significance for novel drug discovery and development. As an alternative to *in vitro* laboratory-based approaches, *in silico* computational methods proposed for DTI prediction have attracted increasing attention in recent years, as they enjoy the high-efficiency and low-cost advantages (Phatak *et al.* 2009). Generally speaking, existing computational methods can be broadly classified into either molecular docking simulation (MDS)-based or machine learning (ML)-based methods (Peska *et al.* 2017).

MDS-based methods aim to simulate the binding process of drugs to their target proteins by predicting the structures of receptor–ligand complexes, where receptors and ligands are target proteins and small drug molecules, respectively. However, their performance in predicting DTIs is subject to the availability of the structure information of targets (Hauser *et al.* 2017). Taking the G-protein-coupled receptors (GPCRs) as an example, few of them can be crystallized as orphan GPCRs, and thereby make it difficult to implement MDS for screening potential interaction sites on the receptor surface to match drug molecules (Ballesteros and Palczewski 2001). To overcome this issue, a variety of ML-based methods have thus been proposed in the light of chemo-genomics, which allows them to identify unknown DTIs through the similarity in the biological information of drugs and targets (Yamanishi *et al.* 2008, Bagherian *et al.* 2021).

Taking advantage of advanced ML techniques, ML-based methods have been widely and successfully applied to DTIs prediction (Wang *et al.* 2014, Luo *et al.* 2017, Wan *et al.* 2019, Bagherian *et al.* 2021). For instance, Wang *et al.* (2014) propose a heterogeneous network-based model, termed TL-HGBI, for DTI prediction. TL-HGBI uses two types of relationships, i.e. drug–disease associations and DTIs, to capture the characteristics of drugs and targets, and an iterative updating algorithm is developed to infer new DTIs. Luo *et al.* (2017) present a novel model with a network integration pipeline, called DTINet, by integrating multiple information to construct a heterogeneous network, and then a compact feature learning method is applied to learn the low-dimensional representation vectors with the topological properties of drugs and targets. Wan *et al.* (2019) develop a nonlinear end-to-end learning model, namely NeoDTI, to learn the network representations of drugs and targets for DTI prediction. NeoDTI first integrates neighborhood information of nodes with information passing, and then a network topology-preserving learning procedure is utilized to extract the representations of drugs and targets. However, ML-based methods typically fail to present satisfactory prediction performance, mainly due to the fact that they rely heavily on the features of given data, which may confuse the classifiers to make correct prediction.

Recently, graph neural network (GNN)-based methods have been widely used in bioinformatics due to better learning of more representative features by considering biological knowledge and topology structure simultaneously (Peng *et al.* 2021, Zhou *et al.* 2021, Li *et al.* 2022). As a representative work in this category, IMCHGAN (Li *et al.* 2022) first adopts a graph attention network to learn the representations for drugs and targets by a specific meta-path, and then employs an attention-based learning algorithm to integrate different meta-path representations as the final features of drugs and targets. MultiDTI (Zhou *et al.* 2021) learns the representations of drugs and targets by multi-modal representation learning, including the region embedding, deep down-sampling residual and the joint presentation modules, on heterogeneous networks. EEG-DTI (Peng *et al.* 2021) incorporates a three-layer graph convolutional network to respectively generate representations of drug and target on the heterogeneous network for DTIs prediction.

When applied to predict DTIs from a given heterogeneous biological information network (HBIN), existing GNN-based models suffer from two disadvantages. First, though they have demonstrated their superior performance in the task of DTI prediction, there is still room for further improvement, as they fall short of adaptively controlling how much information should be aggregated to avoid over-smoothing when learning the feature representations of drugs and targets from HBIN. Second, only a few neural network layers are constructed during aggregation due to the over-smoothing constraint of GNNs, and hence the representative ability of GNN-based computational methods is weakened, thus leading to their unsatisfactory performance for the task of DTI prediction. In particular, the propagation depth, denoted as *k*, allows GNN-based methods to aggregate the information from biomolecules as far as *k*-hop away. However, considering the sparsity of HBIN, it is improper to assign a constant value to *k* for biomolecules located in different regions of HBIN. For example, a larger value of *k* would possibly aggregate excessive information for biomolecules in the dense region, thus leading to over-smoothing. On the other hand, one major advantage of deriving distinguishable feature representations of drugs and targets is to enhance the discriminative ability, which could potentially produce better performance of DTI prediction.

Regarding the solutions proposed to address the over-smoothing issue in the previous studies, they also contain certain limitations. For example, Fast-GCN (Chen *et al.* 2018a) selects a fixed number of graph nodes at each layer to learn their representations. VR-GCN (Chen *et al.* 2018b) considers to reduce the variance on graph node sampling, which can reduce the size of samples, and generate an additional memory cost. Although effective, these methods are not applicable to make an accurate prediction of novel DTIs, as they come at the cost of increased training complexity, and are difficult to generalize in other domain such DTI prediction.

In this work, an improved graph representation learning method, namely iGRLDTI, is proposed to derive high-quality feature representations of drugs and targets over HBIN by addressing the above issue, and thereby achieves promising accuracy for DTI prediction. To this end, iGRLDTI first constructs a complicated HBIN by integrating the molecular structure information of drugs, the sequence information of protein targets, and DTIs. To do so, it is possible for iGRLDTI to collect evidence from different perspectives to support or against the verification of DTIs. After that, it calculates a node-specific propagation depth for each biomolecule in HBIN with a node-dependent local smoothing (NDLS) strategy (Zhang *et al.* 2021). Distinguishable feature representations of drugs and targets can thus be learned with enhanced discriminative power, and they are then incorporated into a Gradient Boosting Decision Tree (GBDT) classifier adopted by iGRLDTI to predict novel DTIs. For the purpose of performance evaluation, a series of extensive experiments have been conducted by comparing iGRLDTI with several state-of-the-art computational methods on the benchmark dataset. Experimental results demonstrate the superior performance of iGRLDTI in terms of several independent evaluation metrics. Besides, our case study also indicates that iGRLDTI considerably alleviates over-smoothing to derive more discriminative feature representations of drugs and targets, and it also provides new insight into the identification of novel DTIs with these distinguishable features.

## 2 Materials and methods

**Figure 1.**
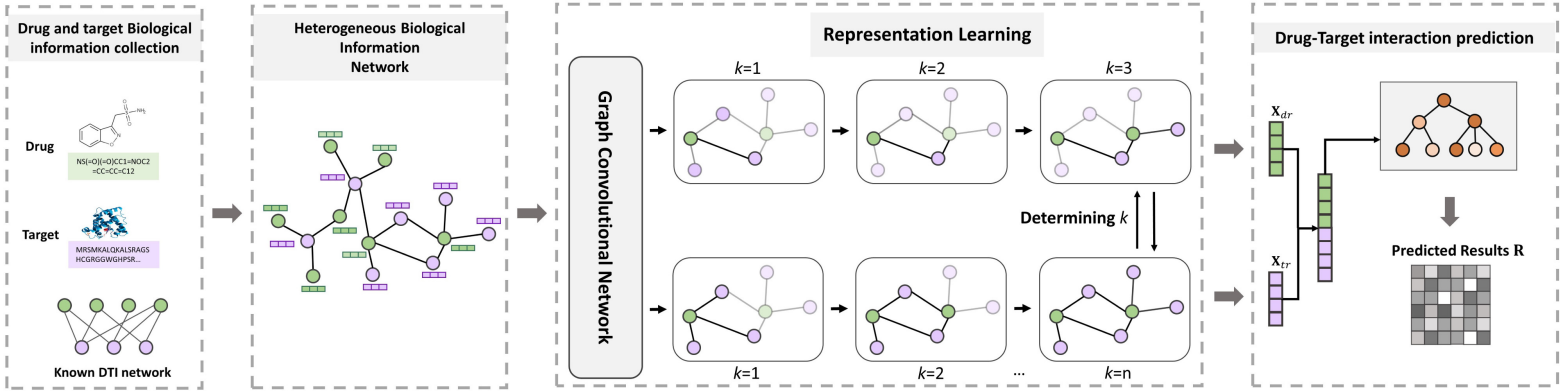
The schematic diagram of iGRLDTI

iGRLDTI is composed of three steps, including HBIN construction, representation learning, and DTI prediction. In particular, the purpose of HBIN construction is to integrate DTIs and the biological knowledge of drugs and targets, such that these different sources of information can be learned in a single context. Given the HBIN, a representation learning process is then performed by iGRLDTI with an NDLS strategy, which allows it to adaptively specify the propagation depth for each biomolecule in HBIN during the learning process. After that, iGRLDTI trains a GBDT classifier with distinguishable feature representations of drugs and targets to complete the prediction task. The overall workflow of iGRLDTI is presented in Fig. 1. Before presenting the details of iGRLDTI, we first introduce the mathematical preliminaries as below.

### 2.1 Mathematical preliminaries

A HBIN is defined as a three-element tuple, i.e. G={V,C,E}, where $V=\{V_{d},V_{t}\}$ denotes a total of |V| biomolecules including drugs ($V_{d}$) and targets ($V_{t}$), **C** is a matrix representing the biological knowledge of drugs and targets, and $E=\{e_{ij}\}$ is composed of all |E| DTIs. Moreover, the number of drugs is $\left|V_{d}\right|$ and that of targets is $\left|V_{t}\right|$. Hence, we have $\left|V_{d}|+|V_{t}|=|V\right|$.

Regarding the biological knowledge of drugs and targets of interest, we employ the molecular structure information of drugs and the sequence information of protein targets to compose **C**. Assuming that $C_{d}\in R^{n\times d}$ and $C_{t}\in R^{m\times d}$ are the respective feature matrices of drugs and targets obtained from their biological knowledge, we have $C={\left[C_{d};C_{t}\right]}^{T}\in R^{(n+m)\times d}$. Given *V* and *E*, a |V|×|V| adjacency matrix of G can be constructed as $A=[a_{ij}]$, where the value of *a_ij_* is 1 if $e_{ij}\in E$ and 0 otherwise. For a |V|×|V| adjacency matrix **A**, *a_ij_* is 0 if both *i* and *j* are drug nodes, and its value will not be changed in the following procedure.

In addition to the above symbols, a |V|×|V| matrix $Q=[q_{ij}]$ is defined to indicate the mutual influence between nodes in given a HBIN when we decide the propagation depth, i.e. *k*, for an arbitrary biomolecule in G during graph representation learning.

### 2.2 HBIN construction

Given all DTIs used to construct G, it is not difficult for us to explicitly compose *V* and *E*, where *V* is composed of 549 kinds of drugs *V_d_* and 424 kinds of targets *V_t_*. Furthermore, all drugs and targets in the DTI data are collected from the DrugBankV3.0 database (Knox *et al.* 2011) and HPRD2009 databases (Keshava Prasad *et al.* 2009), respectively. Regarding their interactions, Luo *et al.* (2017) extract them from the DrugBankV3.0 database, and there are a total of 1923 DTIs. Hence, the only element to be determined in G is **C**, which is calculated as below.

Regarding $C_{d}$, we first collect the Simplified Molecular Input Line Entry System (SMILES) (Weininger 1988) from the DrugBank database (Wishart *et al.* 2018), and then process it with the RDKit tool (Landrum 2013) to obtain $c_{d}$, which is considered as the feature vector of each drug, denoted as $v_{d}\in V_{d}$. Due to $c_{d}$ is high-dimensional, thus, we adopt a reducing auto-encoder strategy (Vincent *et al.* 2008) to obtain a more compact form of $c_{d}\in R^{1\times d}$, here, d is set to 64. Given all $c_{d}$, we are able to obtain $C_{d}={\left[c_{d1};c_{d2};\cdots ;c_{d\left|V_{d}\right|}\right]}^{T}$. In the field of deep learning, it is quite common to apply dimension reduction for achieving a more compact form of embedding vectors. The reducing auto-encoder strategy is adopted to deal with high-dimensional features of drugs and targets in this work, whereas other compared methods make use of different dimension reduction techniques for the same purpose. For example, MultiDTI (Zhou *et al.* 2021) reduces the feature dimensions of targets and drugs adjusting the size of convolution kernel in a deep convolutional neural network. One should note that the reducing auto-encoder strategy could induce information loss when disposing of redundant information (Mohamed *et al.* 2022).

Although there are various sources of biological information used to describe proteins, such as genomic information, protein structures, protein sequences, and Gene Ontology (GO) (Bagherian *et al.* 2021, Pan *et al.* 2022), we simple use the sequence information of protein targets to construct $C_{t}$, as only using protein sequences could yield a more universal performance than other kinds of information about proteins (Guo *et al.* 2008). For each target $v_{t}\in V_{t}$, we first collect its sequence from the STRING database (Szklarczyk *et al.* 2019). Since a protein sequence is a polymer chain composed of 20 different amino acids, the dimension of feature vectors of 3-mers could be as large as 8000 (20^3^), thus consuming more computational resources for further processing. Hence, for the purpose of dimension reduction, a popular way to deal with amino acids is to group them into four categories according to their chemical properties (Shen *et al.* 2007). In particular, these four categories are non-polar amino acids (Glycine, Alanine, Valine, Leucine, Isoleucine, Phenylalanine, Proline), polar and neutral amino acids (Tryptophan, Serine, Tyrosine, Cystine, Methionine, Asparagine, Glutarnine, Threonine), acidic amino acids (Asparagine, Glutamicacid), and basic amino acids (Lysine, Arginine, Histidine). After conversion, the dimension of feature vectors of 3-mers is reduced to 64 (4^3^). One should note that specific amino acids are over-represented only if they are more frequently found in protein sequences. Given all $c_{t}$, we are able to obtain $C_{t}={\left[c_{t1};c_{t2};\cdots ;c_{t\left|V_{t}\right|}\right]}^{T}$. One should note that $c_{t}$ is also a 64-dimensional vector, as there are a total of 64 (4×4×4) possible combinations of 3-mers.

Thus far, we are able to compose **C** with $C_{d}$ and $C_{t}$, and thereby construct G for learning the distinguishable representations of drugs and targets.

### 2.3 Representation learning

For DTI prediction, existing GNN-based computational methods generally learn the feature representations of drugs and targets by simultaneously considering network topology and biological knowledge available in G (Hu *et al.* 2020), and different GNN models are then adopted to achieve this purpose, such as graph attention network (Veličković *et al.* 2018) used by IMCHGAN (Li *et al.* 2022), and graph convolutional network (Kipf and Welling 2017) used by EEG-DTI (Peng *et al.* 2021). However, as the propagation depth, i.e. *k*, of these GNN-based methods increases, the mixture of neighborhood features gathered from biomolecules as far as *k*-hop away drives the output of an GNN model toward a space with less informative features of drugs and targets, resulting in the over-smoothing issue (Huang *et al.* 2020). According to the classical GNN model, the feature representations of drugs and targets at the *k*-th layer, denoted as $X^{\left(k\right)}$, can be obtained by the feed forward propagation a recursive manner. This propagation process can be described as: where $D=[d_{ij}]$ is the diagonal node degree matrix of G, r∈[0,1] is the convolutional coefficient, **W** is the trainable weight matrix at the *k*-th layer, and σ(·) is an activation function. As has been pointed out by Wu *et al.* (2019) and Li *et al.* (2018), the over-smoothing issue is mainly caused by the multiplication of $\bar{A}$ and **X** in Equation (1). To facilitate the derivation of $X^{\left(k\right)}$, we simply let σ(·) and **W** be an identity function and an identity matrix respectively, and then Equation (1) can be rewritten as below. where $X^{\left(0\right)}$ is the initial representation matrix equivalent to **C**. After smoothing $X^{\left(k\right)}$ through a propagation process with infinite depth (k→∞), the final representations of drugs and targets can be obtained as: where ${\bar{A}}^{\left(\infty \right)}=[{\bar{a}}_{ij}^{\left(\infty \right)}]$ is the final adjacency matrix of G, and accordingly ${\bar{a}}_{ij}^{\left(\infty \right)}$ indicates the weight between *v_i_* and *v_j_* ($v_{i},v_{j}\in V$).


(1) $$X^{\left(k\right)}=\sigma (\bar{A}X^{\left(k-1\right)}W^{\left(k-1\right)}),$$

(2) $$\bar{A}=D^{r-1}AD^{-r},$$

(3) $$X^{\left(k\right)}={\bar{A}}^{\left(k\right)}X^{\left(0\right)},$$

(4) $$X^{\left(\infty \right)}={\bar{A}}^{\left(\infty \right)}X^{\left(0\right)},$$

(5) $${\bar{a}}_{ij}^{\left(\infty \right)}=\frac{{\left(d_{ii}+1\right)}^{r}{\left(d_{jj}+1\right)}^{1-r}}{2|E|+|V|},$$

Assuming that $X_{i}$ is the representation vector of *v_i_* and it is also the *i*-th row of **X**, we are interested in measuring the what extent a change in $X_{j}^{\left(0\right)}$ affects $X_{i}^{\left(k\right)}$ when computing **Q**. The value of $q_{ij}^{\left(k\right)}$ can thus be determined with Equation (6).


(6) $$q_{ij}^{\left(k\right)}=\frac{\partial X_{i}{}^{\left(k\right)}}{\partial X_{j}{}^{\left(0\right)}}.$$

Obviously, $Q_{i}^{\left(k\right)}$ denoted as the *i*-th row of $Q^{\left(k\right)}$, indicates the distribution of influence made by other nodes to *v_i_* at the *k*-th layer. Following Zhang *et al.* (2021), we adopt the NDLS strategy to determine the node-specific minimal value of *k* with a distance parameter *ε*, which is an arbitrary small constant to control the smoothing effect. Hence, the definition of NDLS is given as: where $\|\cdot {\|}_{2}$ is the two-norm and $\mathrm{NDLS}(v_{i}^{dr},\varepsilon )>0$. Once we decide the minimal value of *k* for learning $X_{i}$, an average operation is applied to aggregate sufficient neighborhood information within *k*-hops from *v_i_*, and thereby we have the following update rule for $X_{i}$.


(7) $$\text{NDLS}(v_{i},\varepsilon )=\min\{k:\|Q_{i}^{\left(\infty \right)}-Q_{i}^{\left(k\right)}{\|}_{2}<\varepsilon \},$$

(8) $$X_{i}=\frac{1}{\text{NDLS}(v_{i},\varepsilon )+1}\sum_{k=0}^{\text{NDLS}(v_{i},\varepsilon )}X_{i}^{\left(k\right)}.$$

With Equation (8), we are able to obtain the representation vector for each of nodes in G, and **X** can thus be determined. Regarding the matrix **Q**, each of its elements, say *q_ij_*, indicates the mutual influence between nodes *i* and *j*. Even when both *i* and *j* are drug nodes, we also could obtain the value of *q_ij_* with Equation (6).

In particular, iGRLDTI can extract distinguishable representation features by confirming the optimal propagation depth for each node, and further avoiding the over-smoothing phenomenon. Furthermore, the features within *k* hops are aggregated, and then averaged to better capture effectively neighborhood information. In doing so, iGRLDTI pursues higher-order neighbor information while preserving local feature information.

### 2.4 DTI prediction

With the above steps, iGRLDTI is able to extract distinguishable feature representations **X** of drugs and targets. After that, the GBDT classifier (Friedman 2001) is adopted to complete the prediction task with **X**. Specifically, the task of DTI prediction is regarded as a binary classification task under supervised learning. Hence, we prepare a training dataset, denoted as *E*_train_, to build the GBDT classifier based on the representations of drugs and targets. Assuming that *E*_test_ is the testing dataset composed by *N* drug–target pairs with unknown interactions. For an arbitrary drug–target pair, i.e. $<v_{i}\in V_{d},v_{j}\in V_{t}>$ in *E*_test_, we concatenate $X_{i}$ and $X_{j}$ to compose its feature vector $h=[X_{i},X_{j}]$. Moreover, we define r=[r] as a result vector of length *N*, and use it to store the prediction score of each drug–target pair in *E*_test_. One should note that the range of each element in ***r*** is within [0,1]. Obviously, *v_i_* and *v_j_* are more likely to interact if the value of corresponding *r* is larger. The complete procedure of iGRLDTI is described in Algorithm 1.

**Algorithm 1.** The complete procedure of iGRLDTI

**Input:** graph G={V,C,E}; representation sizes: *d*; the number of regression trees: *T*

**Output:** the predicted results matrix **R**

1: Initialization: **R**

2: Extract drug biological features $C_{d}$

3: Extract protein biological features $C_{t}$

4: $C=\left[\begin{array}{c} C_{d}\\ C_{t} \end{array}\right]$

5: $X^{\left(k\right)}={\bar{A}}^{\left(k\right)}C$

6: $q_{ij}^{\left(k\right)}=\frac{\partial X_{i}{}^{\left(k\right)}}{\partial X_{j}{}^{\left(0\right)}},\;Q^{\left(k\right)}=\{q_{ij}^{\left(k\right)}\}$

7: **for**  $v_{i}\in V$  **do**

8:   $\text{NDLS}(v_{i},\varepsilon )=\min\{k:\|Q_{i}^{\left(\infty \right)}-Q_{i}^{\left(k\right)}{\|}_{2}<\varepsilon \}$

9:   $X_{i}=\frac{1}{\text{NDLS}(v_{i},\varepsilon )+1}\sum_{k=0}^{\text{NDLS}(v_{i},\varepsilon )}X_{i}^{\left(k\right)}$

10: **end for**

11: **for**  $<v_{i},v_{j}>$ in *E*_train_  **do**

12:   $h=[X_{i},X_{j}]$

13:   Train the GBDT classifier with ***h*** and *T* as input

14: **end for**

15: //Predict novel DTIs

16: **for**  $<v_{i},v_{j}>$ in *E*_test_  **do**

17:   $h=[X_{i},X_{j}]$

18:   r=GBDT(h)

19: **end for**

20: Return r=[r]

## 3 Results and discussion

### 3.1 Evaluation criteria

To better demonstrate the performance of iGRLDTI, we conduct extensive experiments on the benchmark dataset composed of 549 drugs, 424 targets, and 1923 DTIs (Luo *et al.* 2017). Regarding performance evaluation, AUC and AUPR are used as standardized indicators. In particular, AUC is an area under the receiver operating characteristic (ROC) curve, and AUPR is an area under the precision–recall (PR) curve. Besides, we also adopt the F1 score that is a harmonic mean of Precision and Recall. The calculation of F1 score is given as: where TP and TN are the respective numbers of positive and negative samples predicted to be true, FP and FN are the respective numbers of positive and negative samples predicted to be false. In the experiment, the performance of iGRLDTI is evaluated under a 10-fold cross-validation (CV) scheme. More specifically, we randomly divide the benchmark dataset into 10 folds with equal size. Each fold is alternately selected as the testing data, while the rest are used as the training data. Regarding the generation of negative samples, we randomly pair up drugs and targets whose interactions are not found in the benchmark dataset, and the number of negative samples is equal to that of positive ones.


(9) $$\text{Precision}=\frac{\text{TP}}{\text{TP}+\text{FP}},$$

(10) $$\text{Recall}=\frac{\text{TP}}{\text{TP}+\text{FN}},$$

(11) $$F1-\text{score}=\frac{2\;*\;\text{Precision}\;*\;\text{Recall}}{\text{Precision}+\text{Recall}},$$

### 3.2 Performance comparison

Regarding the performance of iGRLDTI, we have compared it with five state-of-the-art algorithms proposed for DTI prediction, i.e. DTINet (Luo *et al.* 2017), EEG-DTI (Peng *et al.* 2021), NeoDTI (Wan *et al.* 2019), IMCHGAN (Li *et al.* 2022), and MultiDTI (Zhou *et al.* 2021). Experiment results of 10-fold CV are presented in Table 1, and we have compiled the source codes of these methods downloaded from their repositories, and re-evaluated their performances in the same experimental environment for ensuring a fair comparison. Regarding parameter values, we explicitly adopt the default settings recommended in their original work during the training process. We note that iGRLDTI achieves the best performance among all comparing algorithms in terms of AUC and AUPR. On average, iGRLDTI performs better by 3.85%, 0.75%, 1.50%, 3.55%, and 1.20% than DTINet, EEG-DTI, NeoDTI, IMCHGAN, and MultiDTI, respectively. This could be a strong indicator that the proposed representation learning process considerably improves the accuracy of DTI prediction, and iGRLDTI can be a promising tool to identify novel DTIs.

**Table 1.** Comparison with state-of-the-art models on the benchmark dataset.

Metrics	DTINet	EEG-DTI	NeoDTI	IMCHGAN	MultiDTI	iGRLDTI
AUC	0.916	0.953	0.957	0.957	0.961	0.965
AUPR	0.939	0.964	0.945	0.904	0.947	0.967
F1-score	0.091	0.828	0.810	0.892	0.868	0.899

The reasons accountable for the promising performance of iGRLDTI are 2-fold. On the one hand, it employs the biological knowledge of drugs and proteins to enrich the content of HBIN, and then learns their feature vectors from biological knowledge as the initial representations. On the other hand, it adopts the NDLS strategy to decide the node-specific propagation depth during representation learning, thus alleviating the impact of the over-smoothing issue. But for the other comparing algorithms, they are difficult to adjust the depth of neighbor information aggregation for each node, and accordingly, the representations of drugs and targets learned by their GNN models are less discriminative.

Another point worth to note is that some comparing algorithms exhibit different behaviors for their performance in terms of AUC and AUPR. After an in-depth analysis, we find that the main reason for that phenomenon is ascribed to the introduction of the more heterogeneous information. Taking NeoDTI as an example, its AUC performance ranks as the third-best. iGRLDTI only makes use of chemical structures of drugs, protein sequences of targets, and their interactions to compose a HBIN as its input while NeoDTI integrates the structural similarity network of drugs, the sequence similarity network of targets, and different kinds of associations, such as drug–drug interactions, protein–protein interactions, and drug–disease associations, to construct a heterogeneous network as its input. Hence, the main difference lying in the input between iGRLDTI and NeoDTI is that only DTIs are considered by iGRLDTI. Moreover, iGRLDTI still outperforms NeoDTI at 0.8% of AUC, 2.2% of AUPR, and 8.9% of F1-score, suggesting that it may not be necessary to include so many kinds of associations, as the heterogeneous information given by them could degrade the performance by confusing the classifiers to a certain extent. Consequently, we have reason to believe that rather than incorporating more kinds of associations, our work provides an alternative view to improve the accuracy of DTI prediction by alleviating the over-smoothing issue.

Moreover, the DTI prediction problem is more reasonable to be formulated as an imbalanced classification problem in the real case. It is for this reason that additional experiments have been conducted to evaluate the performance of iGRLDTI on the imbalanced dataset, where the ratio between positive and negative samples is set to be 1:10 by following (Wan *et al.* 2019). The experimental results of 10-fold CV are presented in Table 2. Regarding iGRLDTI, we note that it yields the best performance in terms of AUC and AUPR. Its F1-score is ranked as the second best and is only slightly worse by 0.7% than MultiDTI. However, among all evaluation metrics, particular attention should be paid to AUPR, which is a promising indicator in case of imbalanced datasets (Johnson *et al.* 2012). In terms of AUPR, iGRLDTI performs better by 14.1%, 25.5%, 5.9%, 3.8%, and 1.0% than DTINet, EEG-DTI, NeoDTI, IMCHGAN, and MultiDTI, respectively. Hence, we have reason to believe that iGRLDTI is preferred as a promising DTI prediction tool when applied to the imbalance datasets in the real case.

**Table 2.** Comparison with state-of-the-art models under imbalanced samples.

Metrics	DTINet	EEG-DTI	NeoDTI	IMCHGAN	MultiDTI	iGRLDTI
AUC	0.916	0.953	0.946	0.929	0.967	0.986
AUPR	0.786	0.672	0.854	0.603	0.917	0.927
F1-score	0.093	0.813	0.772	0.754	0.828	0.821

### 3.3 Ablation study

**Figure 2.**
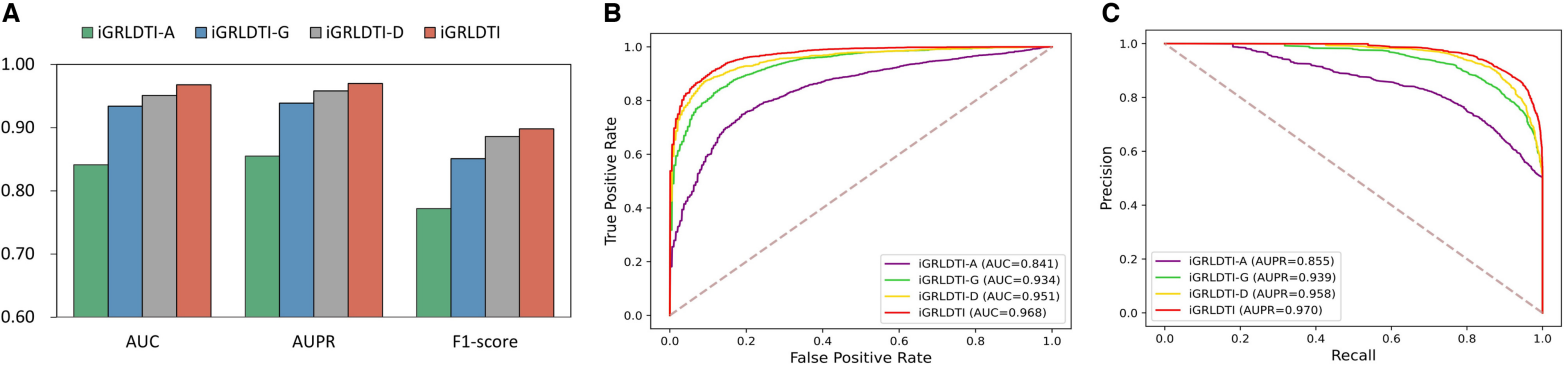
(A) Experimental results of iGRLDTI-A, iGRLDTI-G, and iGRLDTI. (B) The ROC curves are obtained by two variants of iGRLDTI over the benchmark datasets in the ablation study. (C) The PR curves are obtained by two variants of iGRLDTI over the benchmark datasets in the ablation study

To study the impacts of biological knowledge and the over-smoothing issue on the performance of iGRLDTI, we also develop another two variants of iGRLDTI, i.e. iGRLDTI-A and iGRLDTI-G. Specifically, iGRLDTI-A only takes into account the biological knowledge of drugs and targets, i.e. drug molecule structures and protein sequences, while iGRLDTI-G learns the feature representations of drugs and targets based on a classical GNN model as described by Equation (1). Moreover, these two variants also use the GBDT classifier with the same hyper-parameter setting to predict novel DTIs. Experiment results of 10-fold CV are presented in Fig. 2A, and the ROC and PR curves of iGRLDTI-A, iGRLDTI-G, and iGRLDTI are presented in Fig. 2B and C, where several things can be noted.

First, iGRLDTI-A achieves the worst performance when compared with iGRLDTI-G and iGRLDTI. In this regard, only considering the biological knowledge of drugs and targets is difficult to build an accurate prediction model for discovering novel DTIs. Second, iGRLDTI-G presents a better performance against iGRLDTI-A. Under 10-fold CV, iGRLDTI-G achieves an average 9.3% relative gain in AUC and 8.4% in AUPR on the benchmark dataset when compared with iGRLDTI-A. Hence, the aggregation of neighborhood information through the topological structure of HBIN enhances the expressiveness of **X**, which is the representation matrix of drugs and targets. Last, it is noted from Fig. 2A that iGRLDTI outperforms iGRLDTI-G by 3.4%, 3.1%, and 4.6% in terms of AUC, AUPR, and F1-score, and a further improvement is observed from iGRLDTI by addressing the over-smoothing issue. Accordingly, the resulting representations of drugs and targets are more distinguishable than those learned by iGRLDTI-A and iGRLDTI-G.

To investigate the impact of such information loss, a new variant of iGRLDTI, i.e. iGRLDTI-D, is implemented. The only difference between iGRLDTI and iGRLDTI-D is that iGRLDTI-D simply use the one-hot encoding of amino acids, rather than their categories, to compose the feature vectors of 3-mers. The performance of iGRLDTI-D is presented in Fig. 2B and C, and we note that iGRLDTI yields a relative improvement of 1.7% and 1.3% in terms of AUC and AUPR, respectively when compared with iGRLDTI-D. Obviously, the use of amino acid categories allows iGRLDTI to compose the feature vectors of 3-mers in a more compact manner without much redundant information, and accordingly, iGRLDTI performs better than iGRLDTI-D.

### 3.4 Over-smoothing analysis

**Figure 3.**
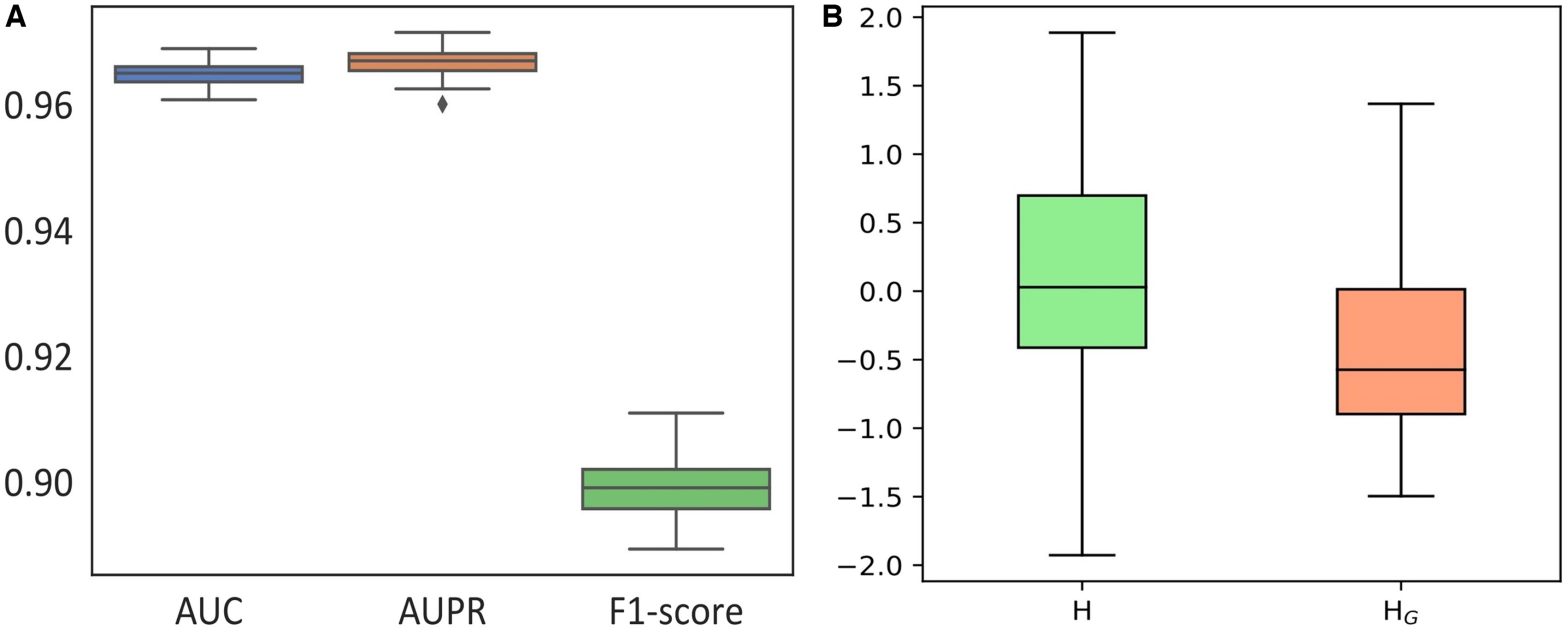
(A) The values of AUC, AUPR, and F1-score by iGRLDTI under 100 rounds of 10-fold cross-validation. (B) Distribution between DB01110 and P54284 representation vectors learned from iGRLDTI and iGRLDTI-G

In the context of deep learning, smoothness is normally used to indicate the similarity across the embedding vectors of nodes. Obviously, less discriminative features of nodes are extracted if their embedding vectors are more similar. When the number of GNN layers increases, node representations become more similar, thus leading to the over-smoothness issue. With no exception in an HBIN, the over-smoothing issue could degrade the performance of DTI prediction. To quantitatively measure the over-smoothing degree, we additionally adopt a frequently used evaluation metric, i.e. Mean Absolute Distance (MAD) (Chen *et al.* 2020), which is defined to compute the average distance between node representations. It is repeatable for the observation in Table 3, and we can now measure the over-smoothing situation by MAD values in the text above. One should note that MAD is proposed to calculate the mean average distance among node representations, and its value is within the range [0, 1]. A smaller MAD score indicates that deep learning models encounter a more severe over-smoothing issue, and thus higher MAD scores can indicate the learned representations are more discriminative. It is noted from Table 3 that the MAD score of iGRLDTI is significantly larger than that of iGRLDTI-G. This could be a strong indicator that for drugs and targets, their representations learned by iGRLDTI exhibit more discriminative features, thus leading the observation in Fig. 3B frequently made across all drug–target pairs. Since iGRLDTI is able to adaptively adjust the propagation depth for each node during representation learning, the impact of the over-smoothing issue is substantially weakened, thus improving the accuracy of DTI prediction.

**Table 3.** Comparison of MAD values between iGRLDTI and iGRLDTI-G with different propagation depths.

	**iGRLDTI(** $10^{-2}$ **)**	**iGRLDTI-G(** $10^{-2}$ **)**					
		1	2	4	10	100	200
MAD	0.399	0.161	0.103	0.102	0.09	0.08	0.06

All numerical values in Table 4 are in the order of $10^{-2}$.

Regarding the propagation depth *k*, we can observe that the MAD values become smaller as *k* value increases, and thus the over-smoothing phenomenon become serious. In Table 3, we note that the MAD value of iGRLDTI is higher when compared with the MAD value for iGRLDTI-G with *k *=* *1. In other words, node representations learned by iGRLDTI-G with *k *=* *1 are more smoothing than those learned by iGRLDTI, which adopts the NDLS strategy to alleviate the over-smoothing issue. The smaller MAD value obtained by iGRLDTI-G with *k *=* *1 is caused by the insufficient information transfer during the message propagation. Since iGRLDTI alleviates the over-smoothing issue by adaptively adjusting the propagation depth for each node during representation learning. In doing so, iGRLDTI can excavate a local-smoothing state of graph node features within HBIN, and further efficiently improve the ability of the model in task of DTI prediction.

### 3.5 Robustness analysis

To evaluate the robustness of iGRLDTI, we repeat 10-fold CV for 100 rounds and presented the average results of AUC, AUPR, and F1-score obtained by iGRLDTI in Table 1, where iGRLDTI still yields the best performance on the benchmark dataset. Moreover, we also draw the box plots in Fig. 3A to show both the summary statistics and the distributions of AUC, AUPR, and F1-score after 100 rounds. Since the variances of AUC, AUPR, and F1-score are 3.72E−06, 4.84E−06, and 2.15E−05, respectively, iGRLDTI also demonstrates its promising performance in terms of robustness.

Moreover, we conduct statistical hypothesis tests to demonstrate the significant difference in the comparison of AUC, AUPR, and F1-score. In particular, we perform the Paired Wilcoxon test by comparing iGRLDTI with other prediction models in terms of AUC, AUPR, and F1-score, and present the results in Table 4. Obviously, iGRLDTI significantly outperforms other prediction models at a confidence level of 95% (*P*-value < .05). This again indicates the superior advantage of iGRLDTI in DTI prediction.

**Table 4.** Comparison of the Paired Wilcoxon test by comparing iGRLDTI with other prediction models.

iGRLDTI	DTINet	EEG-DTI	NeoDTI	IMCHGAN	MultiDTI
*P*-value	0.03662	0.02852	0.03125	0.01242	0.01618

*P*-value <.05 signifies that the results are statistically significant.

### 3.6 Case study

The purpose of our case study is to assess the practical ability of iGRLDTI in terms of identifying unknown DTIs. In the case study, all known DTIs in the benchmark dataset are first taken as positive samples to compose the training dataset, and they are collected from DrugBank V3.0. Regarding the negative samples, we randomly pair up drugs and targets whose interactions are not found in the positive samples. Moreover, in the training dataset, the number of negative samples is the same as that of positive samples. After that, all drug–target pairs that are not found in the training dataset constitute the testing dataset. The cutoff is set as 0.5 to claim predicted DTIs. In other words, a drug–target pair is predicted to be interacted with each other if its prediction score is greater than 0.5. In terms of prediction scores, top 20 pairs in the testing dataset are selected for further validation, and each of them is verified with the latest version of DrugBank, i.e. V5.0. In other words, these verified drug–target pairs are not existed in DrugBank V3.0, but later added into DrugBank V5.0 due to the update of this database (Wishart *et al.* 2018). Following the same procedure as iGRLDTI, top-20 drug–target pairs predicted by each compared model are selected for further investigation in our case study. The top 20 pairs of drugs and targets with the largest prediction scores are presented in Table 5. It is worth noting that the top-20 DTIs pairs can be verified by the latest version DrugBank database (Wishart *et al.* 2018), which means the drug–target pair are not connected when training the iGRLDTI model, they can be predicted by iGRLDTI as candidate DTI and verified by DrugBank database. Consequently, iGRLDTI yields a better performance when compared with other comparing algorithms in discovering unknown DTIs. Taking MultiDTI as an example, only three out of the top 20 pairs have been verified by the DrugBank database, and none of these three verified pairs are ranked in top 5. Besides, we also analyze the performance of iGRLDTI and MultiDTI on the task of discovering DTIs for Zonisamide (ID: DB00909), which is a recommended drug in treating partial seizures (Wilfong and Willmore 2006). Regarding the prediction results, we find that for iGRLDTI, all the five targets predicted to interact with Zonisamide have been verified by the DrugBank database. But for MultiDTI, there are a total of three predicted targets, and none of them could be verified. Hence, this could be a strong indicator that iGRLDTI has a promising performance for discovering novel DTIs when compared with state-of-the-art DTIs prediction algorithms.

**Table 5.** Top 20 predicted results by iGRLDTI.

Rank	Drug ID	Protein ID	Evidence	Rank	Drug ID	Protein ID	Evidence
1	DB00909	O43570	DrugBank	11	DB01268	P17948	DrugBank
2	DB01110	Q14500	DrugBank	12	DB00909	P00918	DrugBank
3	DB00909	Q99250	DrugBank	13	DB00398	P17948	DrugBank
4	DB00594	P19634	DrugBank	14	DB01224	P28335	DrugBank
5	DB01159	P48051	DrugBank	15	DB01268	P09619	DrugBank
6	DB00661	O95180	DrugBank	16	DB00398	P15056	DrugBank
7	DB00594	P19801	DrugBank	17	DB01268	P36888	DrugBank
8	DB01159	O60391	DrugBank	18	DB00398	P04049	DrugBank
9	DB01159	P48549	DrugBank	19	DB01110	Q13936	DrugBank
10	DB00909	O43497	DrugBank	20	DB00909	P21397	DrugBank

Another case study is given to further analyze how iGRLDTI avoids being over-smoothing by comparing its performance with iGRLDTI-G. As mentioned in the section of ablation study, iGRLDTI-G is a variant of iGRLDTI by using a classical GNN model, and hence it is prone to encounter the over-smoothing issue during representation learning. In particular, we note that the interaction between the drug *DB01110* and the target protein *P54284* is successfully identified by iGRLDTI, but not by iGRLDTI-G, where *DB01110* is the drug ID of Miconazole in the DrugBank database, and *P54284* is the uniport ID of Voltage-dependent L-type calcium channel subunit beta-3. Hence, we investigate the prediction scores yielded by iGRLDTI and iGRLDTI-G for this drug–target pair, and find that the prediction score of iGRLDTI, i.e. 0.98, is much larger than that of IGRLDTI-G, i.e. 0.39. In other words, iGRLDTI is more confident to indicate the interaction between *DB01110* and *P54284*, but iGRLDTI-G fails to identify the interaction, as its prediction score is below the cutoff, i.e. 0.5. To validate the solidly of the observation in Fig. 3B, we additionally employed interquartile ranges (IQRs) as a measure of dispersion within the vector elements. A smaller IQR value corresponds to a shorter length of the boxplot, indicating a higher level of similarity among the vector elements, whereas a high boxplot with a larger IQR value hints at the differentiation within the vector elements. Hence, we calculate the average IQR for the feature representations learned from all unknown DTIs using iGRLDTI and iGRLDTI-G, resulting in values of 1.11 ± 0.015 and 0.93 ± 0.019, respectively. Consequently, the observation depicted in Fig. 3B is not an isolated incident but rather a common occurrence.

Assuming that **H** and $H_{G}$ are the concatenated representation vectors of *DB01110* and *P54284* learned by iGRLDTI and iGRLDTI-G, respectively, we present their boxplots in Fig. 3B to visualize the difference between **H** and $H_{G}$ from the distribution perspective. The height of a boxplot, to some extent, indicates the difference among the elements in the corresponding vector. In particular, a short boxplot means that all the elements in a vector are similar to each other, whereas a tall boxplot hints at the differentiation within the vector elements. It is observed from Fig. 3B that the difference in the elements of $H_{G}$ is much smaller than that of **H**. This could be a strong indicator that the representation vectors learned by iGRLDTI-G still suffer the over-smoothing issue, and it is for this reason that iGRLDTI-G fails to predict the DTI between *DB01110* and *P54284*. Since iGRLDTI is able to learn more distinguishable representations by alleviating the over-smoothness from an alternative view, the accuracy of DTI prediction can thus be improved.

In sum, these case studies again demonstrate the promising performance of iGRLDTI in discovering new DTIs with more distinguishable representations, and hence it is believed that iGRLDTI could be a useful tool to identify novel DTIs.

## 4 Conclusion

In this article, an improved graph representation learning method, namely iGRLDTI, is developed to discover novel DTIs over HBIN. To this end, iGRLDTI first constructs an HBIN by integrating the biological knowledge of drugs and targets with their interactions. Then, iGRLDTI adopts an NDLS strategy to adaptively decide the propagation depth during representation learning, thus significantly enhancing the discriminative ability of their representations by alleviating over-smoothness. Finally, iGRLDTI employs the GBDT classifier to achieve the DTI prediction task. Experimental results demonstrate that iGRLDTI yields a superior performance under 10-fold CV when compared with several state-of-the-art prediction algorithms, and furthermore, our case studies indicate that iGRLDTI is able to learn more distinguishable representations of drugs and targets, and it is a useful tool to identify novel DTIs.

There are two reasons contributing to the superior performance of iGRLDTI. On the one hand, the construction of HBIN allows iGRLDTI to learn the representations of drugs and targets from multiple views. Due to the rich information carried by HBIN, the task of DTI prediction can be achieved by iGRLDTI in a more effective manner. On the other hand, with the NDLS strategy, iGRLDTI is able to determine the node-specific propagation depth for each biomolecule in HBIN. Consequently, it adaptively controls how much neighborhood information should be gathered to avoid over-smoothness during representation learning.

Besides, we also note several limitations of iGRLDTI. On the one hand, a simple weighted averaging method is applied to update $X_{i}$, and it is difficult for us to differentiate the significance of $X_{i}^{\left(k\right)}$ at the *k*-th layer. One the other hand, not all drugs and targets are able to provide necessary biological knowledge especially for those newly discovered, and hence the prediction performance of iGRLDTI is weakened for drugs and targets without sufficient biological knowledge.

Regarding the future work, we would like to unfold it from four aspects. First, we intend to improve the performance of iGRLDTI by proposing solutions to address its limitations. Second, we are interested in evaluate the generalization ability of iGRLDTI by applying it to other prediction problems, such as protein–protein interactions prediction and drug–drug interaction prediction. Third, we would like to investigate the performance of iGRLDTI by integrating more kinds of associations, as it is a challenging task to fully exploit the heterogeneous information for improved performance of DTI prediction. Last, we also would like to explore the interpretability of iGRLDTI in order to provide interpretable prediction results (Schulte-Sasse *et al.* 2021).

## Data Availability

The real data underlying this article are available from https://github.com/stevejobws/iGRLDTI.
